# Mantle cell lymphoma of the colon: case report and literature review

**DOI:** 10.1093/jscr/rjaf347

**Published:** 2025-05-30

**Authors:** Gregory Crisafulli, Pasha Shenasan, Aakash Trivedi, Nawras Radwan, Abraham El-Sedfy

**Affiliations:** Department of Surgery, St. Joseph’s University Medical Center, 703 Main St, Paterson, NJ 07503, United States; Department of Surgery, St. Joseph’s University Medical Center, 703 Main St, Paterson, NJ 07503, United States; Department of Surgery, St. Joseph’s University Medical Center, 703 Main St, Paterson, NJ 07503, United States; Department of Surgery, St. Joseph’s University Medical Center, 703 Main St, Paterson, NJ 07503, United States; Department of Surgery, St. Joseph’s University Medical Center, 703 Main St, Paterson, NJ 07503, United States

**Keywords:** gastrointestinal lymphoma, B cell lymphoma, mantle cell lymphoma, cecal mass, hemicolectomy

## Abstract

Primary extranodal lymphoma is a rare presentation of B-cell non-Hodgkin’s lymphoma (NHL), most commonly found in the gastrointestinal tract. Mantle cell lymphoma (MCL), a rare and aggressive subtype, accounts for only 2.5% of all lymphoid neoplasms and is less commonly localized in the colon. We present a 79-year-old man with abdominal pain and nausea. Imaging revealed cecal wall thickening with ileocolic intussusception. Colonoscopy identified a circumferential cecal mass, necessitating robotic-assisted right hemicolectomy. Final pathology confirmed MCL. The patient recovered well without complications. Colorectal MCL is a rare and aggressive NHL variant requiring a multidisciplinary approach. Surgery plays a crucial role in managing complications and localized disease. Given the lack of standardized treatment protocols, further research is needed to optimize therapeutic strategies and long-term outcomes.

## Introduction

Mantle cell lymphoma (MCL) is a rare and aggressive B-cell non-Hodgkin lymphoma (NHL), comprising ⁓2.5% of all lymphoid neoplasms in the United States. While extranodal involvement is common, primary gastrointestinal (GI) MCL is rare, with colorectal involvement particularly uncommon. Among extranodal lymphomas, the GI tract is the most affected site, though the colon and rectum are less frequently involved compared to the stomach or small intestine. Colorectal MCL often presents with vague symptoms such as abdominal pain, weight loss, GI bleeding, or, in rare cases, bowel obstruction. Intussusception in adults is uncommon, and when associated with malignancy, it is linked to primary or metastatic neoplasms. MCL diagnosis relies on histopathological and immunohistochemical evaluation, with overexpression of cyclin D1 and markers like CD20, CD5, and BCL-2. Due to the aggressive nature of MCL and the absence of standardized treatment protocols, management involves surgery, chemotherapy, and targeted therapies. This case report discusses a 79-year-old male with primary colorectal MCL manifesting as ileocolic intussusception requiring surgical intervention, emphasizing the importance of early recognition and timely management.

## Case description

**Figure 1:**
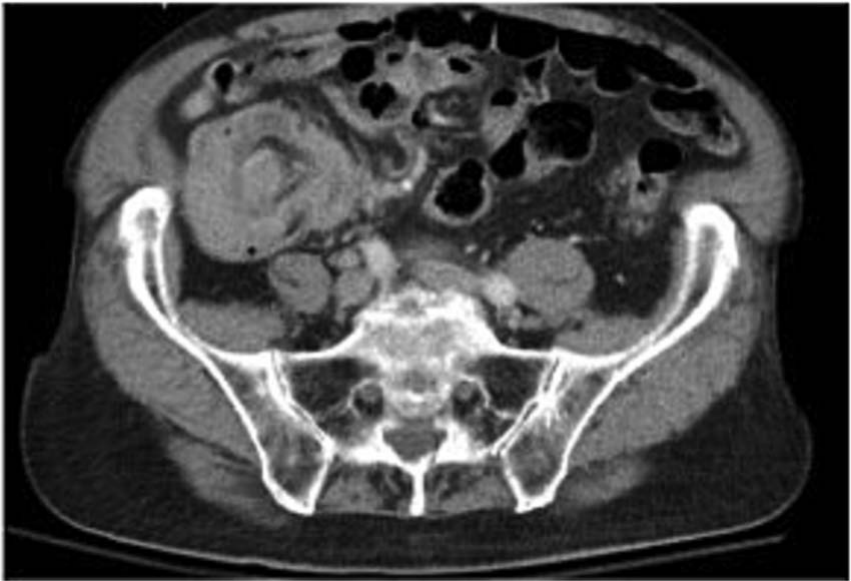
Axial view of the ileocecal intussusception.

**Figure 2:**
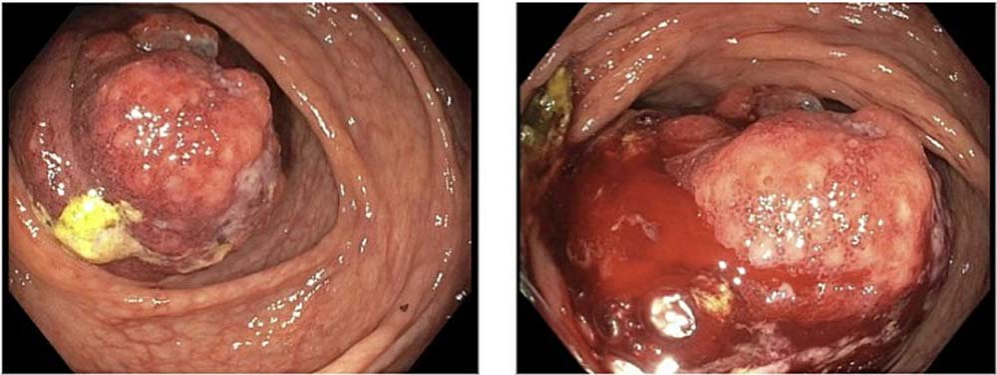
Visualization of cecal mass during colonoscopy.

A 79-year-old Spanish-speaking male with hypertension, hyperlipidemia, and benign prostatic hyperplasia presented with intermittent, bilateral lower quadrant abdominal pain and nausea for 3 days. The pain was described as aching, non-radiating, and 6/10 in intensity. He had regular bowel movements and tolerated a normal diet. His past surgical history included a right inguinal hernia repair, and he had a normal colonoscopy 5 years prior, except for benign polyps. Lab work, including a carcinoembryonic antigen (CEA) test (0.5 ng/mL), was normal. A computerized tomography (CT) scan revealed a thickened cecum and ileocecal intussusception (Fig. 1). Gastroenterology performed a colonoscopy with cold forceps biopsies, revealing a large, infiltrative, polypoid mass in the cecum (Fig. 2). Pending pathology, the patient underwent a robotic-assisted right hemicolectomy due to impending bowel obstruction, followed by a functional end-to-end anastomosis. He tolerated the procedure well and was scheduled to begin chemotherapy with Bendamustine and Rituximab, selected due to his advanced age and frailty.

Histological examination revealed lymphomatous polyposis in the small bowel and colon. Immunohistochemistry showed strong positivity for CD20, BCL-2, CD43, Cyclin D1, and focal positivity for CD5. Seventeen pericolonic lymph nodes exhibited partial involvement. SOX-11 was faintly positive, while p53 expression was wild-type. The lymphoma cells were negative for CD10, BCL-6, and IgD. Ki-67 staining indicated a proliferation index of 30%–50%. Fluorescence in-situ hybridization (FISH) revealed no evidence of CCND1-IGH [translocation t(11,14)] or BCL2-IGH [translocation t(14,18)] gene rearrangements, no evidence of trisomy 11 or gain of 11q, and no evidence of BCL6 (3q27) breakpoint translocation.

## Discussion

MCL is characterized by the clonal proliferation of malignant B-lymphocytes from the mantle zone of lymphoid follicles [1]. Primary extranodal lymphomas, which affect organs without prior lymph node involvement, are rare [2]. The GI tract is the most common extranodal site, accounting for 10%–30% of cases, though colorectal involvement is uncommon. Colorectal lymphomas show a male predominance with a male-to-female ratio of >2:1 and a median age of 60 [1]. Adult intussusception, though rare (2–3 cases per 1 000 000), is associated with malignancy in 30%–60% of cases in the small and large bowel, respectively [3].

Colorectal MCL lacks well-established risk factors but may share risks with other extranodal NHLs, such as immunosuppression, viral infections (human immunodeficiency virus, hepatitis C), family history, and radiation exposure [2]. The disease may also be influenced by infections affecting the gut microbiota, though no specific associations have been found [4]. MCL is typically characterized by cyclin D1 overexpression, resulting from a translocation between chromosomes 11 and 14, dysregulating the cell cycle [5]. Despite cyclin D1 overexpression in this case, no translocation t(11;14) was detected. Immunohistochemical staining showed positivity for CD20, CD5, BCL-2, and cyclin D1, consistent with MCL [1, 6, 7]. SOX11, an oncogene involved in MCL, regulates tumor cell migration, adhesion, and proliferation [8].

Colorectal MCL presents with nonspecific symptoms like abdominal pain, blood per rectum, fatigue, fevers, and weight loss [6, 7]. In this case, the patient only presented with abdominal pain and nausea. Given these nonspecific symptoms, diagnostic imaging is essential. CT scans help identify masses, lymphadenopathy, tumor size, and invasion depth. In this case, imaging revealed colonic intussusception, leading to surgical consultation. Similarly, Smith *et al*. [1] reported a case of MCL presenting with intermittent abdominal pain and colonic intussusception. Intussusception due to malignant lymphoma accounts for over 10% of colonic intussusceptions [3]. Colonoscopy and biopsy are key for visualization and diagnosis. In this case, CEA levels were normal, making it an unreliable marker for diagnosis [1, 6].

The definitive diagnosis of colorectal MCL requires biopsy and immunohistochemistry, with additional molecular studies like flow cytometry and FISH to differentiate variants. Treatment is challenging due to MCL's rarity and aggressiveness. The National Comprehensive Cancer Network (NCCN) Guidelines recommend chemotherapy regimens such as RDHA (rituximab, dexamethasone, cytarabine, platinum-based agents) or R-CHOP (rituximab, cyclophosphamide, doxorubicin, prednisone), though R-CHOP may be poorly tolerated in some patients [9].

Surgical resection was indicated due to ileocecal intussusception caused by lymphoma. Kella *et al.* [10] reported successful resection and treatment of MCL causing ileocecal intussusception, emphasizing a multidisciplinary approach. Monoclonal antibodies combined with multi-agent chemotherapy have shown 80%–95% response rates in MCL [10]. Bandaru *et al.* [11] found that surgery followed by chemotherapy improves outcomes in localized colonic lymphomas, with some patients achieving remission after R-CHOP therapy. However, the median survival for MCL remains 3–5 years [1].

The MCL International Prognostic Index (MIPI) [12] assesses prognosis based on age, performance status, lactate dehydrogenase levels, and leukocyte count. Additional markers like TP53 and Ki-67 improve prognostic accuracy, classifying patients into low, intermediate, or high-risk categories [13]. In the absence of standardized treatment regimens, early diagnosis and multidisciplinary management are critical. Surgical intervention may offer therapeutic benefits, especially for GI complications like intussusception. Further studies are needed to establish optimal treatments and improve long-term outcomes [14].

## Conclusion

Colorectal MCL is a rare and challenging form of NHL requiring a nuanced diagnostic and treatment approach. This case emphasizes the critical role of early diagnostic tools, like CT and immunohistochemistry, in detecting and confirming MCL. The nonspecific symptoms and aggressive course of MCL necessitate a multidisciplinary approach, with surgery playing a pivotal role in cases involving GI complications. Surgery can address immediate complications like intussusception and serve as a curative measure in localized disease. The lack of standardized treatment protocols highlights the need for further research to refine prognostic models and improve therapeutic outcomes.
